# Baby Steps – a structured group education programme with accompanying mobile web application designed to promote physical activity in women with a history of gestational diabetes: study protocol for a randomised controlled trial

**DOI:** 10.1186/s13063-018-3067-8

**Published:** 2018-12-12

**Authors:** Nithya Sukumar, Helen Dallosso, Ponnusamy Saravanan, Tom Yates, Carol Telling, Karen Shorthose, Alison Northern, Sally Schreder, Christopher Brough, Laura J. Gray, Melanie J. Davies, Kamlesh Khunti

**Affiliations:** 1 Population, Evidence & Technologies, Division of Health Sciences, Warwick Medical School, University of Warwick, Coventry, CV4 7AL UK; 2 Leicester Diabetes Centre, University Hospitals of Leicester, Leicester General Hospital, Leicester, LE5 4PR UK; 3 Academic Centre for Diabetes and Endocrinology, George Eliot Hospital, Nuneaton, CV10 7DJ UK; 4 Diabetes Research Centre, College of Medicine, Biological Sciences and Psychology, University of Leicester, Leicester, LE5 4PW UK; 5 NIHR Leicester Biomedical Research Centre, University of Leicester, Leicester, UK; 6 Research & Development Department, George Eliot Hospital, Nuneaton, CV10 7DJ UK; 7 Department of Health Sciences, College of Life Sciences, University of Leicester, Leicester, LE1 7RH UK; 8 NIHR Collaboration for Leadership in Applied Health Research and Care - East Midlands, University of Leicester, Leicester, UK

**Keywords:** Randomised controlled trial, Gestational diabetes, Self-management, Patient education, Diabetes prevention

## Abstract

**Background:**

A diagnosis of gestational diabetes (GDM) is associated with an over sevenfold increase in the risk of developing type 2 diabetes (T2D), while among parous women with T2D, up to 30% have a history of GDM. Lifestyle interventions have been shown to reduce the risk of incident T2D in adults with impaired glucose tolerance, including in women with a history of GDM. The aim of this study is to establish whether a group self-management education programme, supported by a mobile web application, can improve levels of physical activity at 12 months in women who have had GDM.

**Methods:**

The study is a randomised controlled trial with follow-up at 6 and 12 months. Primary outcome is change in objectively measured average daily physical activity at 12 months. Secondary outcomes include lipid profile, blood pressure, glycated haemoglobin, obesity, smoking and alcohol status, self-reported physical activity, anxiety, depression and quality of life. Participants are recruited from maternity and diabetes departments in hospital trusts in two sites in the UK. Women aged > 18 years, with a diagnosis of GDM during any pregnancy in the previous 60 months are eligible. Participants need to have a good understanding of written and verbal English, be able to give informed consent and have access to a smart-phone. Women who are pregnant or have type 1 or type 2 diabetes are not eligible. In total, 290 participants will be recruited and randomly assigned, with stratification for age and ethnicity, to either the control group, receiving usual care, or the intervention group who are invited to participate in the Baby Steps programme. This comprises a group education programme and access to a mobile web application which provides an education component and interacts with a wrist-worn activity monitor providing automated messages, setting challenges and encouraging motivation.

**Discussion:**

If effective, the Baby Steps programme could be translated into a primary care-based intervention that women with GDM are referred to in the postnatal period. This could help them make lifestyle changes that could reduce their future risk of T2D.

**Trial registration:**

ISRCTN, ISRCTN17299860. Registered on 5 April 2017.

**Electronic supplementary material:**

The online version of this article (10.1186/s13063-018-3067-8) contains supplementary material, which is available to authorized users.

## Background

### Maternal outcomes following gestational diabetes

Around 700,000 women give birth in England and Wales each year; up to 5% of them have a diagnosis of diabetes in pregnancy [1]. Among women with diabetes in pregnancy, gestational diabetes mellitus (GDM; described as abnormal glucose tolerance which first develops or is recognised during pregnancy) constitutes around 85% of cases, with the remainder due to pre-existing type 1 diabetes (T1D) or type 2 diabetes (T2D). The aetiology of GDM is not completely known but is due in part to the inability of the maternal pancreas to secrete sufficient insulin to cope with pregnancy-induced insulin resistance in susceptible women. The incidence of GDM is on the rise, in part due to higher rates of obesity in the general population, including in women of childbearing age [2–4]. Estimates suggest that lifestyle factors such as obesity, smoking, unhealthy diet and physical inactivity may explain around 50% of the incidence of GDM [5].

A diagnosis of GDM is associated with adverse outcomes for both the mother and her affected offspring. These women have higher risks of pre-eclampsia, Caesarean sections and an over sevenfold increase in the risk of developing T2D, with the highest incidence occurring within five years of the index pregnancy [6–9]. Among parous women who have a diagnosis of T2D, ≤ 30% have a previous history of GDM, which means that pregnancy and the postnatal period within the first five years present a ‘golden opportunity’ to intervene and alter the natural course of a disease [10, 11]. Additionally, women with a history of GDM have 2–3 times higher incidence of hypertension and ischaemic heart disease so any intervention provided in the postnatal period has the added potential of reducing cardiovascular disease risk [8].

Fetal macrosomia (defined as birthweight > 4–4.5 kg) and large for gestational age (LGA) (defined as birthweight > 2 SD greater than mean or > 90th centile after controlling for age and sex) are two of the most common and serious offspring outcomes of GDM in pregnancy. Babies born to GDM mothers are 4–7 times likely to be macrosomic [12, 13]. Other perinatal complications which are also associated with hyperglycaemia during pregnancy include shoulder dystocia and birth injuries, neonatal hypoglycaemia and respiratory distress syndrome [14]. In the long term, offspring of women with GDM are at increased risk of obesity, hypertension, hyperlipidaemia and glucose intolerance starting from childhood and early adolescence thereby perpetuating the cycle [15–17].

### Role of lifestyle interventions to reduce T2D

There is evidence from observational studies that an active lifestyle (including increase in physical activity time and reduction in sedentary time) is associated with weight loss, improved glucose tolerance and lower risk of progression to T2D in women with a history of GDM [18, 19]. The Diabetes Prevention Programme showed, with a randomised controlled trial (RCT) design, that intensive lifestyle interventions reduced the risk of incident T2D in adults with impaired glucose tolerance (IGT) by around 40–60% [20, 21] and, in a subgroup of women with a history of GDM, by 53% and 35% over three and ten years, respectively, compared to standard care [22, 23]. Other trials have shown decreases in rates of pre-diabetes, weight, waist circumference and lipid levels [24–26]. Interestingly, treatment with metformin has been shown to confer no additional benefit to lifestyle interventions, presumably due to adherence issues and less weight loss in the first year of treatment when compared to the latter [22].

However, there are several barriers to lifestyle interventions in postpartum women with recent GDM, including lack of time, balancing work and family demands, and lack of childcare [27, 28]. Recently, a web-based lifestyle intervention program in 75 women in Boston, USA was deemed to be feasible and associated with significant weight reduction (3.3 kg below the control group) and higher likelihood of being below pre-pregnancy weight at 12 months postpartum [29]. The program particularly recommended gradually increasing physical activity to 150 min per week, including resistance training and making healthier dietary choices.

While most of the abovementioned trials have suggested that a combination of exercise, healthy diet and weight loss or weight maintenance protects against diabetes in postpartum women, there is a lack of evidence about the independent effect of physical activity in reducing progression from GDM to T2D, particularly in high-risk populations such as minority ethnic groups. Indeed, in observational series, increase in moderate to vigorous physical activity, independent of body mass index (BMI), was associated with reduction in the risk of T2D in women after pregnancy [18]. Among non-pregnant adults, every 2000 step per day change from baseline to 12 months is associated with an additional 8% decrease in the cardiovascular event rate [30].

The ongoing and completed intervention programmes for women with a history of GDM have been conducted largely in women of white American, Australian or European background [19, 23, 31, 32] and black, Hispanic and mixed minority American [24, 33–35]. Therefore, there is a paucity of information about the feasibility and effectiveness of similar programmes for women in the UK, particularly in those of South Asian ethnicity. It is known that up to one in five South Asian adults globally have T2D and the diagnosis is 4–5 times more common in South Asian adults in the UK, compared to a white reference group [36, 37].

Our study will therefore be novel in developing and testing an intervention that meets the cultural and social needs of women in a catchment area in that has a large multi-ethnic population (estimated to constitute 25–60% of the women diagnosed with GDM in the two research sites).

## Methods/Design

### Aims and objectives

1. To assess the effectiveness of a group education programme and accompanying online web support (Baby Steps programme) in improving objectively measured physical activity at 12 months in participants with a history of GDM compared to usual care.
2. To assess the effect of the programme on other risk factors including lipid profile, blood pressure, resting heart rate, glycated haemoglobin (HbA1c), obesity, smoking and alcohol status, self-reported physical activity, anxiety, depression, quality of life, fruit and vegetable intake compared to usual care.
3. To assess the acceptability, uptake and feasibility of delivering the programme to women who are at high risk of developing T2D, including those from ethnic minority backgrounds.

### Study design and setting

**Fig. 1:**
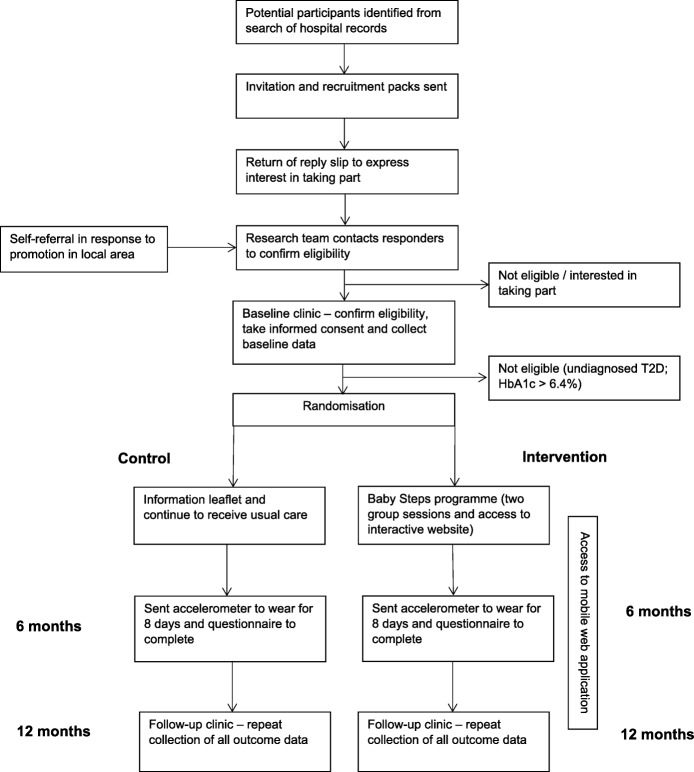
Consort flow diagram

**Fig. 2:**
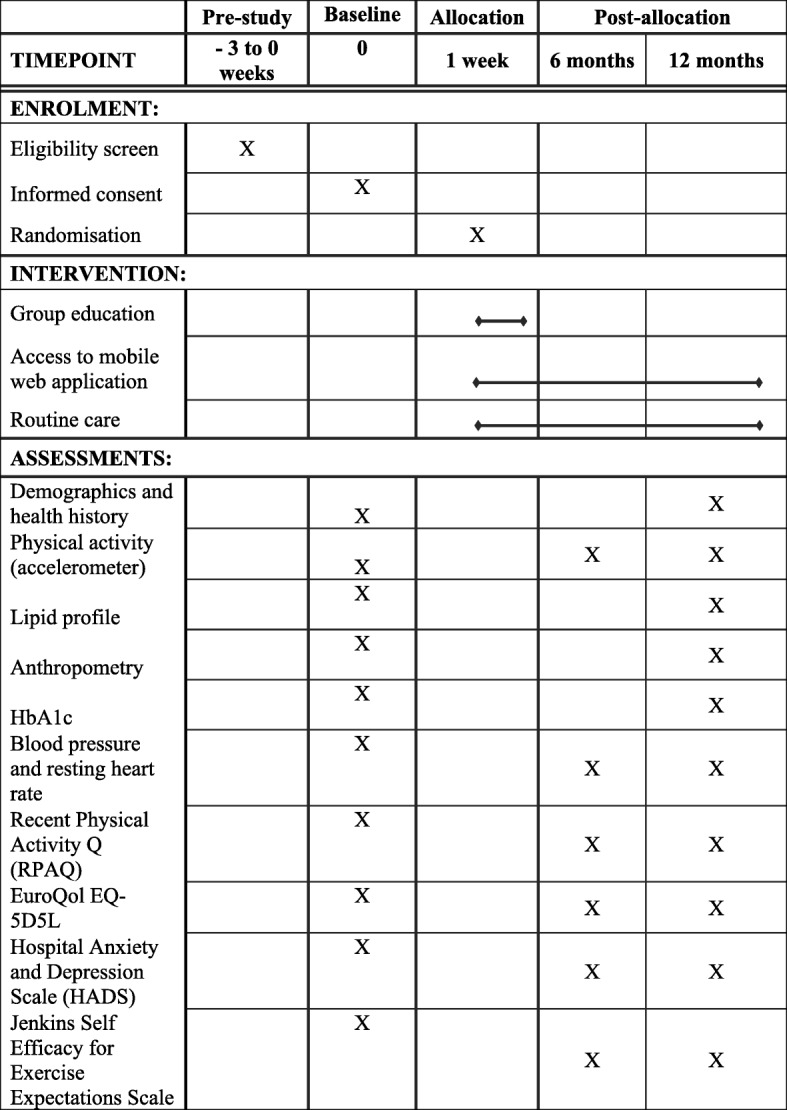
Schedule of enrolment, interventions and assessments as per SPIRIT 2013

The study is a two-group (1:1), parallel RCT of women who have had GDM during any pregnancy up to 60 months before the point of recruitment and are therefore at risk of developing T2D. Figure 1 describes the flow of participants through the study. The RCT is being conducted in two sites in the UK, namely University Hospitals of Leicester NHS Trust (serving the catchment area of Leicester City and the county of Leicestershire) and George Eliot Hospital NHS Trust (serving the catchment area of North Warwickshire). The protocol was written in accordance with the Standard Protocol Items: Recommendations for Interventional Trials (SPIRIT) guidelines (see Spirit Fig. 2 and the SPIRIT Checklist, which is available as Additional file 1). The study is sponsored by the University of Leicester and ethical approval was granted by the East Midlands – Derby Research Ethics Committee. The study was prospectively registered (ISRCTN 17299860).

### Recruitment and informed consent

Potential participants are identified from hospital databases (both maternity and diabetes departments) and are sent a recruitment pack containing an invitation letter, a brief information leaflet detailing the study and a reply slip. Reply slips are returned to the local study team. A member of the study team then contacts the respondent by telephone to screen them and arrange a baseline visit to confirm their eligibility, take their informed consent and collect the baseline data. Following the telephone call, they are sent a letter confirming the details of the appointment and a copy of the full Patient Information Leaflet (PIL). This is sent with sufficient time to enable them to have at least 24 h to read the PIL. A reminder invitation is sent to non-responders 4–6 weeks after the initial invitation. Potential participants who do not respond to the reminder invitation within four weeks may be called by a member of the direct care team. The purpose of the call is not to coerce the women to take part but to confirm they have received the invitation and to find out whether they understand the information they have been sent and whether they have any questions. There is also the opportunity for women to self-refer in response to posters in community venues and publicity in the local media.

### Eligibility criteria

The inclusion criteria are as follows:

- women aged ≥ 18 years, with a diagnosis of GDM during any pregnancy resulting in delivery in the last 60 months;
- willing and able to attend the clinic and education sessions;
- able to speak and read English sufficiently to give informed consent and follow the education programme.

The exclusion criteria are as follows:

- currently pregnant;
- diagnosis of T1D or T2D;
- cancer (not in remission);
- severe diagnosed mental illness (e.g. schizophrenia, bipolar);
- previous surgical or medical intervention to treat obesity;
- lack of access to Internet;
- participated in another clinical intervention study in the previous 12 weeks.

Women who consent to join the study and are identified as a result of the baseline blood tests as having undiagnosed T2D (HbA1c > 6.4%) are withdrawn from the study and referred back to their general practitioner.

### Data collection clinics

Primary and secondary outcome data are collected at baseline and 12 months (when participants attend data collection clinics) and some are also collected at six months (paperwork is sent and returned by post). Baseline data are not collected until eligibility has been confirmed and the participant has given written informed consent.

Clinics are held at the two hospital sites or at local community venues and are staffed by appropriately trained research nurses and healthcare assistants. All clinical measures are carried out in accordance with study-specific standard operating procedures (SOPs) in place at the two sites and standard calibrated equipment is used. Participants are given a £15 gift voucher for every data collection clinic or education session they attend as a contribution towards childcare costs. If the session is held in a venue where childcare is available, the cost is paid by the study budget and the participant does not receive a voucher.

### Randomisation and blinding

Study identification numbers are assigned sequentially and participants are individually randomised (1:1) stratified by age (< 30 years; ≥ 30 years) and ethnicity (White European; other) using a variable block size after their baseline assessment. The randomisation schedule was developed by an independent statistician and allocation of randomisation is carried out by a researcher based in Leicester who is independent of the team. Randomisation determines which group the participant is allocated to (control or intervention). After randomisation, participants in both groups are sent a letter informing them of the outcome of the randomisation. In addition, intervention participants are contacted by telephone to discuss the dates available for them to attend the Baby Steps programme. They are then sent a letter confirming the venue and dates and times of the programme. Since the intervention is a group self-management programme, participants and the local research teams cannot be blinded to the randomisation. The staff analysing the accelerometer data to derive the primary outcome are blinded to allocation.

### Treatment regimens

#### Control group

Control group participants are sent an information booklet about the prevention of diabetes.

#### Intervention group

Participants randomised to the intervention group are invited to participate in the Baby Steps programme. This has two components: a group-based structured education programme and a secure mobile web application which provides access to information and learning tools to supplement the group programme.

The group programme is based on Let’s Prevent Diabetes and Walking Away from Diabetes, group education programmes which have been shown to be effective for people at increased risk of developing T2D [38, 39]. They are based on robust theoretical frameworks and the philosophy is based on patient empowerment [40–42]. The Baby Steps programme comprises two group sessions that are each 3 h long and are delivered approximately two weeks apart. A total of 8–10 people are booked to attend each programme and are given the opportunity to bring a friend or member of the family if they wish. Session 1 focuses on the background to GDM and being at risk of T2D, physical activity and other modifiable risk factors and goal setting (Table 1). The second session revisits physical activity and reviews progress on goals, barriers and solutions and then covers diet and food choices with goal setting relating to reducing risk of developing T2D. The programme is delivered in a facilitative style that encourages participation with the use of reflective questioning and problem-solving activities to promote engagement. The education sessions are delivered at the two hospital sites as well as at local community venues. The target is to attend within two months of recruitment. However, a pragmatic approach is taken and this is not always possible. A sensitivity analysis will be performed to take this into account.

**Table 1:** Contents of the Baby Steps group education programme

Session 1	Session 2
Introduction and housekeeping (5 min)	Welcome back (5 min)
Your story (25 min)	Sharing stories (30 min)
Blood glucose (20 min)	Revisiting physical activity (15 min)
How could being at risk of diabetes affect my health? (15 min)	Weight management (30 min)
Risk story (25 min)	Food choices (45 min)
Being more active (70 min)	Planning to reduce my risk (30 min)
Reflections (5 min)	Questions and future care (10 min)
TOTAL - 2 h 45 min (+ 15 min break) = 3 h	TOTAL - 2 h 45 min (+ 15 min break) = 3 h

During Session 1, participants are given a secure link to a mobile web application which has been developed to accompany the programme. The application has two main functions: first, to provide an interactive education component to follow on from the group component; and second, to motivate the user into becoming more active. It contains a variety of resources of different formats (e.g. video animations, expert videos, interactive activities and quizzes) which supplement the messages delivered during the group sessions. In addition, there are booster sessions (released monthly for nine months) which contain further information and challenges. Participants are given a wrist-worn activity monitor that can connect to the website using a human application programming interface (API). Regular automated messages are sent in relation to goal setting, goals achieved and the setting of new challenges with daily updates and motivational messages. Participants can opt to join a group who can use the website to share their challenges and experiences with their peers. More information on the application is provided in Additional file 2.

Development of the group education programme followed an iterative pathway comprising various stages of design, testing and refining the programme [43]. It was developed by a multidisciplinary team with substantial co-production from patient and public involvement (PPI) and other stakeholder groups (including midwives and maternity care assistants). Four meetings were held with stakeholders (two with women who had previously had GDM and two with healthcare professionals). The discussions were led by an independent and experienced qualitative researcher from the University of Leicester and informed on content of the programme, the online resource and practical issues such as the number of sessions, ideal venues and times of the day, and whether the session should be limited to women. The interactive website is hosted on a safe secure server and was developed in collaboration with a web designer with healthcare professionals and patient groups involved at all stages. An iterative pathway similar to that used in the development of the group programme was followed.

A small team of facilitators has been trained in each site. They are either appropriately skilled lay people or healthcare professionals. They attended a two-day training programme at the beginning of the study. The first day covered the facilitator skills needed and the theories and models underpinning the programme. The second day covered the curriculum content and ensured they were familiar with the teaching resources used in its delivery. Facilitators were given a curriculum and set of teaching resources and delivered a ‘have a go’ session to volunteer patients before the study started. These practice sessions were observed by a member of the training team and feedback and further training was provided as needed. Continued mentoring and peer support are provided to the facilitators and self-reflection and peer reflection after every session is encouraged.

### Outcome measures

#### Demographics and medical history

The participant’s age, ethnicity, smoking and alcohol status, and number of children are recorded. Details of any relevant history of disease and medications and first-degree family history of diabetes are recorded. Figure 2 summarises the outcome data which will be collected at each study visit.

#### Primary outcome

The primary outcome measure is change in objectively measured physical activity from baseline to 12 months using the GENEActiv wrist-worn tri-axial accelerometer (GENEActiv model 1.1, ActivInsights Ltd., Cambridgeshire, UK) with a dynamic range of ± 8 *g*, where *g* is equal to the Earth’s gravitational pull. Participants are asked to wear the GENEActiv accelerometer on their non-dominant wrist for eight consecutive days (24 h), wearing the monitor from the date of the assessment visit or from a specified date when sent in the six-month postal follow-up. The accelerometer is initialised to collect data at 100 Hz. An appropriately trained individual instructs the participant on correct placement of the monitor. Participants are asked to complete a log while wearing the accelerometer to provide their waking hours and wear time information. Participants are given a prepaid envelope to return the accelerometer and log book once completed. Accelerometer data will be calibrated and analysed according to best practice procedures through the Lifestyle Theme of the NIHR Leicester Biomedical Research Centre. In brief, data will be processed and calibrated using a bespoke open source package in R (GGIR http://cran.r-project.org according to criteria previously described [44–46]. Data will be included if participants have one or more valid days of data, with a valid day defined as at least 16 h of wear time. The primary outcome is defined a priori as overall movement intensity as quantified by the Euclidean Norm minus 1 *g* (ENMO) method. In addition, time asleep, sleep quality, time in sedentary behaviour, light-intensity physical activity and moderate to vigorous physical activity will also be derived using validated algorithms and thresholds.

#### Secondary outcomes

##### Clinical and anthropometric measures

Blood pressure and resting pulse rate are measured after the participant has been sitting for 5 min. Three measurements are made; the first measurement will be excluded when calculating the mean. Body weight (kg) and height (m) are measured and used to calculate BMI (weight in kg/m^2^). Waist circumference (cm) is measured at approximately 1 cm above the iliac crest and hip circumference (cm) at the widest area around the gluteus maximus.

##### Blood tests

Venous blood samples for analysis of non-fasting HbA1c and full lipid profile (total cholesterol, LDL cholesterol, HDL cholesterol and triglycerides) are collected at baseline and 12 months by trained personnel. They are analysed in accredited laboratories at University Hospitals of Leicester NHS Trust or at George Eliot Hospital NHS Trust in accordance with their SOPS and are destroyed after analysis. All laboratory results are reviewed and the reports signed by the Principle Investigator at each site or an individual approved by the Chief Investigator in the Delegation of Authority log. The results are recorded in the clinical record form (CRF) and identified as normal, abnormal but not clinically significant, or abnormal and clinically significant. In case of the latter, the eligibility of the participant to continue on the study is reviewed.

Participants may optionally consent to provide blood samples of serum, plasma and whole blood, which will be stored indefinitely for future ethically approved research. At the end of the study, the samples from George Eliot Hospital will be transported to University Hospitals of Leicester using an accredited courier service and stored in a Human Tissue Act licensed laboratory in line with all relevant agreements in place.

##### Questionnaire data

Participants are asked to complete the following questionnaires at baseline, 6 and 12 months. They are given in person at the baseline and 12 months clinics and sent by post at 6 months.

1. Recent Physical Activity Questionnaire (RPAQ): the RPAQ is designed to explore day-to-day physical activity levels in the previous four weeks. The questionnaire comprises three sections: (1) physical activity patterns in and around the house; (2) travel to work and work activities; and (3) recreational activities. RPAQ has reasonable validity for measuring total physical activity levels [47, 48].
2. Health-Related Quality of Life EuroQoL (EQ-5D-5 L): the EQ-5D-5 L assesses health-related quality of life and provides useful data for health economic analyses. It is a validated measure of health status and has five quality of life dimensions (mobility, self-care, usual activities, pain/discomfort and anxiety/depression) which are all coded in the range of 1–5 [49].
3. Jenkins self-efficacy for exercise expectations scale: this validated self-efficacy scale measures ability to exercise when considering nine barriers. These are weather, boredom, pain, exercising alone, lack of enjoyment, busyness, tiredness, stress and depression [50].
4. Hospital Anxiety and Depression Scale (HADS): HADS is a validated scale measuring the severity of symptoms of anxiety and depression. It comprises 14 statements of which seven relate to anxiety and seven relate to depression [49]. Each statement has an option of four responses scored in the range of 0–3. Upon completion the scores selected are totalled and reported for anxiety and depression individually.
5. Fruit and Vegetable intake questionnaire: the Five-A-Day Consumption and Evaluation Tool (FACET), which is recommended by the Department of Health, is used as a measure of fruit and vegetable intake [51] .

### Sample size

The primary outcome is based on increasing total physical activity as quantified by the Euclidean norm minus one (ENMO) method measured in milligravity units (m*g*). This is the main measure of activity derived from the GENEActiv monitor. In order to detect a minimum clinically significant difference of 2.1 m*g*, which is equivalent to an overall increase in physical activity volume of approximately 30 min of light walking at 4 km/h, assuming a standard deviation of 5.3 m*g* [52], a power of 80% and significance level of 5%, the sample size requires 202 participants. To allow for 20% loss to follow-up and 10% non-compliance of the GENEActiv monitor, we will need to recruit 290 participants (145 in each arm).

### Statistical analysis

Descriptive characteristics at baseline will be summarised by treatment arm. Numbers (with percentages) for binary and categorical variables and means (and standard deviations) or medians (with lower and upper quartiles) as appropriate for continuous variables will be presented. Preliminary graphical and tabular presentations of the data will be inspected for the correct statistical modelling assumptions.

The primary analyses will use a complete case population. For the primary outcome (change from baseline in total physical activity), treatment arms will be compared using linear regression with a binary indicator for randomisation group as the explanatory variable, terms for the stratification factors as confounders, and adjustment for the change in accelerometer wear time and baseline total physical activity. Sensitivity analyses will include a per-protocol analysis (defined as attending at least one of the two group education sessions, i.e. those randomised to intervention but not attending either group session will be excluded) and an intention-to-treat analysis where missing data will be imputed using multiple imputations. Interaction effects will be fitted between intervention arm and age (< median vs ≥ median), and ethnicity (White European vs other). If the interaction term is statistically significant then stratified analyses will be performed for that factor using the same model as the primary analyses. Secondary outcomes will be analysed using similar methods as the main analysis, with an appropriate model selected dependent on the distribution of the outcome.

The results of all comparative analyses will be presented with 95% confidence intervals and statistical significance for main effects will be assessed at the 5% level. All *p* values shown will be two-sided. Statistical significance for interaction effects will be assessed at the 10% level.

### Qualitative interviews

A sub-sample of participants in the intervention arm will be invited to take part in a qualitative interview about their experience of attending the Baby Steps programme and, in particular, of using the mobile web application that is part of the programme. Participants will be selected so that both those who do and those who do not engage with the application are interviewed. The interviews will include a discussion on whether the programme meets the social and cultural needs of the women. If the programme is shown to be effective in achieving our primary and secondary outcomes, then this qualitative information may be used to make revisions to the content or mode of delivery of the programme before it is offered to women outside a trial setting.

Soon after attending the 12 month follow-up clinic, selected participants will be invited to take part in a focus group, face-to-face interview or telephone interview (format will be the choice of the participant). Informed consent will obtained by the interviewer before the interview starts. Interviews will be digitally recorded unless the participant does not consent to the recording in which case notes will be made. Interviews will be carried out by an experienced researcher from the University of Leicester following an approved topic guide and then transcribed by a transcription service approved by the sponsor. A purposive sampling strategy will be used to ensure that a cross-section of participants is selected (for example, women from both sites, women who did and did not engage with the application) and it is anticipated that about 15–20 women will be interviewed. Interview data will be analysed through thematic analysis where information will be put into themes and a codebook produced, placing important emphasis on letting the themes emerge from the empirical data. This is the basis of grounded theory and thus there will be an explicit focus on open coding and development of themes as data is collected. Although themes will be based on the initial topic guide, data collection and data analysis will be flexible in nature and adopt a constant comparative approach. To aid structure in analysis because transcripts and qualitative data can be voluminous, research software packages such as Nvivo 10 will be used.

### Data management and monitoring

The Chief Investigator is responsible for ensuring that the study is conducted in full conformity with the current revision of the Declaration of Helsinki and with the ICH Guidelines for Good Clinical Practice (CPMP/ICH/135/95, July 1996). The participants’ anonymity is maintained at all points. Participants are identified only by an ID number and their initials on the CRF and any electronic database. All documents and patient identifiable information are stored securely in locked cabinets and on a password-protected computer or in off-site archiving rooms (according to the sponsor’s SOPs). The study complies with the general data protection regulation which requires data to be anonymised or pseudo-anonymised as soon as it is practical to do so. All data are entered on a validated electronic database which only includes the participant’s ID number and is stored for ten years and then archived according to the sponsor’s SOP. Any identifiable personal data will be permanently deleted from the servers once the final report has been submitted or within five years if the participant has agreed to be contacted for other studies.

As this is a minimal risk study, a Data Monitoring Committee has not been convened. Serious adverse events (SAE) are monitored and reported in line with requirements. An internal group meets every month to review recruitment rate, drop out, issues concerning delivery of the intervention and SAEs. A quarterly report on progress is submitted to the funder.

## Discussion

The National Institute for Health and Care Excellence (NICE) guidelines on diabetes in pregnancy recommend that women who have been diagnosed with GDM are offered a fasting plasma glucose or HbA1c test at 6–13 weeks postnatally, given lifestyle advice (including weight control, diet and exercise) and offered an annual HbA1c test [1]. It also recommends that women with an HbA1c of 39–47 mmol/mol (5.7% and 6.4%) are advised that they are at a high risk of developing T2D and should be offered advice, guidance and interventions in line with available guidelines to prevent T2D. The American Diabetes Association guidelines also recognises the high risk of T2D in this group and recommends women with GDM to have a 75 g oral glucose tolerance test (OGTT) at 4–12 weeks postpartum followed up by additional testing every 1–3 years if the OGTT is normal [53].

Primary care provision of postnatal care to this group is disappointing with annual rates of long-term follow-up of GDM in primary care only around 20% [54]. There is a need to improve the postnatal monitoring of women who have had GDM as well as to provide innovative ways to reduce progression from GDM to T2D.

The Baby Steps programme will aim to address these issues in a RCT with the primary outcome of improving physical activity levels at 12 months. The latter has been previously shown to be independently associated with reduction in risk of T2D and cardiovascular events in high-risk individuals [18, 30]. This robust programme has been developed following a large element of PPI work with input provided by women who have had GDM as well as healthcare professionals who work with this patient group. It is designed to accommodate the family and work commitments of women who have young children and the qualitative interview component of the study will evaluate the perception of the programme by service users who have different levels of engagement.

An important aspect of the programme is the mobile health technology which complements the group education sessions and is being used increasingly in several disciplines in medicine. Smart phones and wearable devices have the potential to improve public health and have shown some success in health behaviour change interventions [55–57]. These new technologies could shift the need from intensive face-to-face intervention towards an interactive, self-directed, personalised and cost-effective tool for women following a diagnosis of GDM. Mobile technology has been used in the management of GDM and the present study will add to this by investigating whether it has a role in the prevention of T2D in women with a history of GDM [58, 59].

The Baby Steps programme, if shown to be effective, could be delivered in a variety of settings, including primary care, to high-risk women from multi-ethnic populations and help reduce incidence of future T2D. The trial is restricted to women who are able to speak English, which limits our ability to meet all the language and cultural needs of a population with ethnic minorities. However, our team has a good track record of delivering similar lifestyle interventions in other languages. Therefore, if the findings from the study and qualitative interviews are positive, it may be possible to conduct the sessions in other languages in future to increase uptake among ethnic minority populations and target women with the highest risk of T2D.

### Trial status

Recruitment started on 5 April 2017 and is ongoing.

### Protocol version

The current protocol version is Version 5; 25 May 2018. Four substantial amendments have been approved. Amendment 1 (before recruitment started) involved a change in the randomisation procedure (from an online software system to manual allocation using a randomisation schedule). Amendment 2 involved making telephone calls to non-responders. Amendment 3 involved a change in eligibility criteria (from recruited within 36 months of most recent delivery to recruited within 60 months of any delivery). Amendment 4 involved carrying out qualitative interviews with participants from the intervention arm.

## Additional files

Additional file 1: SPIRIT 2013 Checklist: Recommended items to address in a clinical trial protocol and related documents*. (DOC 120 kb)

Additional file 2: Baby Steps Mobile Web Application. (PDF 327 kb)

## Abbreviations

API: Human application programming interface
CRF: Clinical record form
ENMO: Euclidean Norm minus 1 *g*
EQ-5D-5 L: Health Related Quality of Life EuroQoL
FACET: Five-A-Day Consumption and Evaluation Tool
GDM: Gestational diabetes
HADS: Hospital Anxiety and Depression Scale
HDL: High-density lipoprotein
IGT: Impaired glucose tolerance
LDL: Low-density lipoprotein
LGA: Large for gestational age
NICE: National Institute for Health and Care Excellence
PIL: Patient information leaflet
PPI: Patient and public involvement
RCT: Randomised controlled trial
RPAQ: Recent Physical Activity Questionnaire
SAE: Serious adverse event
SOP: Standard operating procedure
T1D: Type 1 diabetes
T2D: Type 2 diabetes
