# Supplementary material for: Baby Steps – a structured group education programme with accompanying mobile web application designed to promote physical activity in women with a history of gestational diabetes: study protocol for a randomised controlled trial
Source: Trials. 2018 Dec 12;19:682. doi: 10.1186/s13063-018-3067-8 (PMC6292087; doi:10.1186/s13063-018-3067-8)
Supplement: Supplementary file 2 — Baby Steps Mobile Web Application. (PDF 327 kb) [file 13063_2018_3067_MOESM2_ESM.pdf]

Baby Steps Mobile Web Application

Participants are given a secure login to the application when they attend the first of the two group education sessions. The application can be accessed from a smart phone or tablet and when they register they are given the opportunity to form a group with the women they attended the session with. They are also given a wrist worn activity monitor that can connect to the website using a human application programming interface.

The application has five parts:

Steps

This has three sections

- Steps – this section keeps a record of the number of steps they have done and provides bar charts and graphs of daily and weekly values.
- Challenges – the participant can set themselves daily targets and they are informed what percentage of their target they reach or how often they reach the target
- Achievements – motivational and congratulatory messages are sent

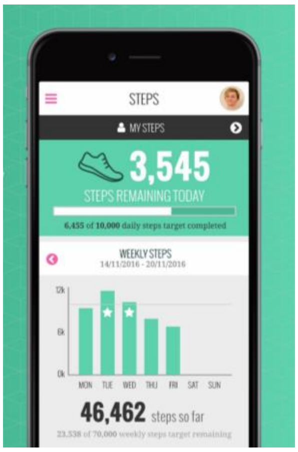

Booster Sessions

These are released monthly for 9 months and cover the following topics

Month 1 – A Balanced Diet
Month 2 – Calories in vs calories out
Month 3 – Health Snacks and Drinks
Month 4 – Get standing
Month 5 – Keeping up your activity
Month 6 – Reducing stress
Month 7 – Healthy living for a health hear
Month 8 – Importance of Strength Training
Month 9 - Graduation

The booster sessions contain a number of resources including

- Animations or interviews. These are 4 to 5 minutes long and are accompanied by a narration with the option of having a written transcript.
- Interactive games and multiple choice quizzes
- Short presentations by researchers and health care professionals

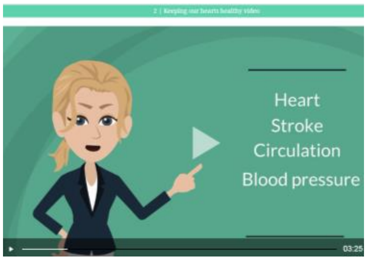

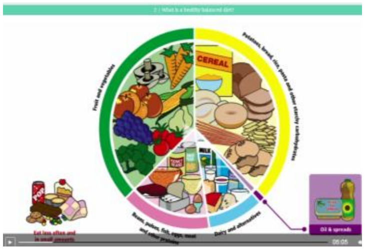

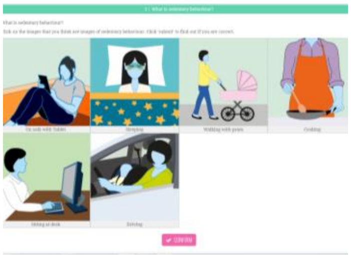

Chat line

Participants are able to chat globally with everyone who has registered with the application and on a group basis with the women who attended the group programme with them

Babysteps Admin As a whole you have so far recorded over 6 million steps, which is about 3000 miles. Basically it is like walking across America!! Well done everyone, you are doing great. [illegible]

Reply (/app/chat/view/global_chat/172) | 0 comments | 9 Feb at 10:13:37

[redacted] Really struggling with steps this week ... and it's only my first week 😢 little one is poorly and we haven't been able to get out and it was my first week back at work this week and my job although in a Children's nursery is office based as the manager 😩 roll on next week when hopefully my little one is better and we can do more stepping!! Hope everyone else is reaching their goals this week!

Reply (/app/chat/view/global_chat/170) | 1 comment | 2 Feb at 19:37:09

[redacted] Hi everyone, i have decided to take part in Mymarathon in order to fundraise for the british heart foundation, this means i shall be running 26.6 miles throughout the month of May, the research they carry out and the support they offer to patients and families is fantastic and i can honestly say that as I'm a nurse and work with heart patients myself so see the difference they make daily, I would really appreciate it if you would all take a minute to have a look at my fundraising page and possibly make a small donation [illegible] thanks in advance https://mymarathon18.everydayhero.com/u[redacted]

Reply (/app/chat/view/global_chat/222) | 4 comments | 27 Apr at 15:00:27

Health Trackers

These sections can be used by the participant to record information on themselves and in this way monitor their health and lifestyle. The information they record is either lifestyle information (body weight and shape, healthy eating and smoking) or information they obtain after visiting the GP or practice nurse (HbA1c or cholesterol levels). The health trackers can be used to monitor changes and make action plans

Educational reading

This contains background information on the following topics

- What does ‘at risk of type 2 diabetes’ mean?
- Healthy food choices
- Become more active
- Reducing your weight

The sections are written for the lay reader and include tables, diagrams and images.

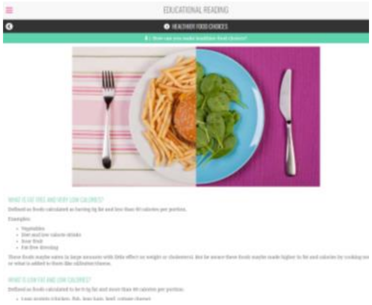

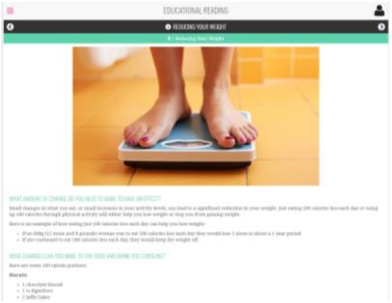

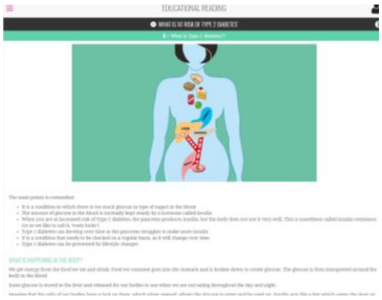

Finally there are two filmed testimonials from women who have had gestational diabetes women and a number of short presentations from various researchers and health care professionals. These are 2 to 3 minutes long and cover the following topics

- What is gestational diabetes?
- Type 2 diabetes
- Diet
- Goal setting
- Reducing sedentary behaviour
- Relapse
